# Beneficial Effect of Olive Leaf Extract as an Adjunct to Standard Antifungal Therapy in Treating *Candida*-Related Oral Diseases

**DOI:** 10.3390/ijms26178193

**Published:** 2025-08-23

**Authors:** Maja Kinkela Devčić, Irena Glažar, Igor Pasković, Daniela Kovačević-Pavičić, Josip Peradinovic, Ivana Munitic, Sunčana Simonić-Kocijan

**Affiliations:** 1 Clinic of Dental Medicine, Clinical Hospital Center Rijeka, 51000 Rijeka, Croatia; majakinkela@yahoo.com; 2 Department of Oral Medicine, Faculty of Dental Medicine, University of Rijeka, 51000 Rijeka, Croatia; irena.glazar@fdmri.uniri.hr; 3 Institute of Agriculture and Tourism, 52440 Poreč, Croatia; paskovic@iptpo.hr; 4 Department of Prosthodontics, Faculty of Dental Medicine, University of Rijeka, 51000 Rijeka, Croatia; daniela.kovacevic@fdmri.uniri.hr; 5 Faculty of Biotechnology and Drug Development, University of Rijeka, 51000 Rijeka, Croatia; josip.peradinovic@biotech.uniri.hr (J.P.); ivana.munitic@biotech.uniri.hr (I.M.)

**Keywords:** antifungal activity, *Candida* spp., olive leaf extract

## Abstract

The aim of this study was to evaluate whether combined administration of olive leaf extract (OLE) with standard antifungal therapy—nystatin (NYS) or miconazole (MIC) could be a more efficient alternative in reducing the number of *Candida* colonies, the presence of oral signs and symptoms and changes in salivary IL-17A level compared to standard therapy alone. The study included 59 subjects with a positive microbiological *Candida* colony number greater than 600 CFU/mL and at least one oral sign or symptom present. Subjects were randomly divided into four groups depending on applied therapy: OLE + NYS group (n = 15), OLE + MIC group (n = 15), NYS group (n = 14), MIC group (n = 15). Therapy duration and clinical monitoring were standardized across all groups. There was no significant difference between the tested groups in *Candida* spp. colony number or salivary IL-17A levels. In the OLE + NYS group, a significant increase in salivation rate was observed, while a significant decrease in tongue burning was reported in the OLE + MIC group. A significant reduction in burning of the oral mucosa and tongue was observed in the MIC group. No significant differences were found in other clinical signs or symptoms among treatment groups. OLE, as an adjunct to standard antifungal therapy, did not significantly reduce *Candida* spp. colony number or salivary IL-17A levels. However, in combination with NYS it increased salivation rate, while in combination with miconazole, it significantly decreased tongue burning. Both symptoms are common clinical findings in oral *Candida*-related disease and suggest that OLE may have supportive potential in the clinical management of these conditions. Further research is needed to explore its potential therapeutic benefits on oral health.

## 1. Introduction

*Candida* species (*Candida* spp.) is a part of the normal oral flora [1]. However, it may cause opportunistic infections influenced by various factors that alter the oral environment and affect the resistance of the oral mucosa such as host immune status, antibiotic use, smoking, age, pregnancy, oral hygiene, and dental prosthetic appliance wearing [2,3,4]. *Candida albicans* appears to be the most common species, followed by *Candida tropicalis*, *Candida glabrata*, *Candida parapsilosis*, and *Candida krusei* [5]. *Candida* spp. may cause various forms of infections, from superficial to systemic conditions [3,4]. Despite the development of new antifungal drugs and research into alternative approaches in treating oral fungal infections [6,7,8], miconazole and nystatin remain the drugs of choice primarily because of their clearly defined usefulness and low prices [9].

The increasing use of antifungal agents has led to the emergence of resistant *Candida* strains, which cause difficulties in the treatment of fungal infections in the oral cavity. Therefore, many researchers are working to find new potential herbal therapeutics that may be a safer alternative in the treatment of oral diseases such as phenolic compounds from olive leaf extract [10]. A combination of standard antifungal therapy and natural products is another way to increase the effectiveness of antifungal drugs and reduce their potential toxic effects. Given these challenges, natural compounds are increasingly considered as supportive therapies alongside standard antifungals, aiming to enhance efficacy and reduce adverse effects. Among natural products, olive leaf extract is a promising therapeutic tool for the treatment of oral infections [11,12]. Many potentially bioactive substances in olive leaves can have antioxidant, antimicrobial, antihypertensive, antiviral, anti-inflammatory, hypoglycemic, neuroprotective, and anticancer properties [13,14,15,16,17,18,19,20].

Interleukin-17A (IL-17A) is one of the key mediators of protection against microorganisms and has a specific role in protection against *Candida* spp. [21,22]. IL-17A and IL-17F induce the production of antimicrobial peptides (defensins), regulate the antifungal response through the regulation of pro-inflammatory cytokines (IL-6, G-CSF, GM-CSF) and chemokines (CXCL1, CXCL2, CXCL5) and enhance surface neutrophil infiltration mucosa [23,24,25,26,27].

The aim of the study was to evaluate whether olive leaf extract used as an adjunct to conventional antifungal therapy influences oral *Candida* species colony count number, oral signs and symptoms, and salivary IL-17A level.

## 2. Results

The study included 59 subjects (n = 59), divided into four groups: 15 (25.4%) were treated with MIC, 14 (23.7%) with NYS, 15 (25.4%) with MIC + OLE, and 15 (25.4%) with NYS + OLE. Regarding gender, 16 (27.1%) were men and 43 (72.88%) women. The median age of the respondents is 71 years (interquartile range from 63 to 80 years) with a range from 22 to 90 years. There are no significant differences in age and gender in relation to the applied therapy (Table 1).

Comparing *Candida* spp. colony number across treatment groups, no significant reduction of colony number was observed with the addition of OLE to NYS and MIC therapy (Table 2).

Clinical examination showed no significant differences in oral clinical signs and subjective symptoms between groups receiving MIC vs. MIC + OLE, or NYS vs. NYS + OLE therapy (Table 3).

Assessed using the Visual Analog Scale (VAS), a significant reduction in tongue burning, was observed on day 7 in the MIC and MIC + OLE groups (*p* < 0.05), while in the NYS group, the reduction was significant from day 3 onwards (*p* < 0.05). Additionally, a significant reduction in oral mucosa burning, also evaluated through VAS scoring, was observed in the MIC group on day 7 (*p* < 0.05).

Salivation significantly increased on day 7 compared to day 0 only in the NYS + OLE group (*p* < 0.05), while no significant changes were observed in other groups (Table 4).

Analysis of salivary IL-17A levels before and after therapy showed no statistically significant differences in the reduction of IL-17A values in subjects who received MIC vs. MIC + OLE and NYS vs. NYS + OLE (Table 5).

## 3. Discussion

*Candida* spp. is a highly adaptable microorganism that can cause infection in various conditions and various places in the host’s organism [1,2]. In healthy individuals, it exists as a commensal organism, but under favorable conditions, such as immunosuppression, or systemic diseases it can become pathogenic, leading to opportunistic infections. The growing prevalence of chronic diseases, including diabetes, malignancies, and autoimmune disorders, contributes to an increased incidence of oral *Candida* infections. Additionally, aging populations, prolonged medication use, and the presence of removable prosthetic appliances further predispose individuals to *Candida* infections [1,2,3,4,5].

The frequent use of antifungal drugs has led to rising concerns about resistance among *Candida* spp. reducing the effectiveness of standard therapies and leading to more frequent negative effects which further complicate clinical management [28]. Because of that, there is a growing need to explore alternative treatments for fungal infections of the oral cavity. This has driven the search for alternative antifungal strategies, including natural plant-derived compounds with potential antimicrobial properties. Given the historical use of medicinal plants in treating infections, olive leaf extract (OLE) has emerged as a promising candidate for adjunctive antifungal therapy due to its rich composition of phenolic compounds. The beneficial effects of OLE are commonly associated with its phenolic compounds, particularly oleuropein, hydroxytyrosol, and other flavonoids, which exhibit antimicrobial, anti-inflammatory, and antioxidant properties. However, the chemical composition of OLE can vary significantly depending on factors such as cultivar, geographic origin, agricultural practices, and the season of leaf harvest, which contributes to its complex and diverse biological activity [19].

Different types of *Candida* species can be isolated from the oral cavity. The most common is *C. albicans* [28,29]. Identification of the correct *Candida* spp. is crucial since different species have different abilities to cause infection and can cause differences in a patient’s response to standard treatment protocols [30]. In addition to *C. albicans*, commonly isolated species from the oral cavity include *C. tropicalis*, *C. krusei*, *C. dubliniensis* and *C. glabrata*, while in some patients it is not unusual to isolate two or more different species at the same time. This complicates treatment, often requiring combination therapy or alternative approaches [10]. Exploring adjunctive therapies such as OLE could be beneficial in enhancing the efficacy of conventional antifungal drugs.

Because of the increasing interest in plant-based antifungal agents, the aim of this study was to assess the potential of OLE as an adjunct to conventional therapy. Although previous research has shown that phenolic compounds in OLE exhibit antifungal activity by inhibiting cell growth and damaging the fungal cell membrane, however, the mechanism of the antimicrobial effect of oleuropein has not been fully clarified [31]. Some authors believe that the orthodiphenolic system (catechol) in the molecular structure of oleuropein is responsible for this effect [12]. Zorić et al. [32], proved in their research that oleuropein causes a decrease in the total content of ergosterol in the cell membrane of *C. albicans*, which could be involved in the mechanism of antifungal action. Our previous research [33] has demonstrated the antifungal potential of OLE in in vitro conditions, demonstrating a dose-dependent inhibitory effect on *Candida* spp. Based on these findings, the aim of the present study was to assess whether OLE, as an adjunct to conventional therapy could enhance antifungal efficacy in vivo. While the in vitro results suggested promising antifungal properties, our current findings indicate that OLE did not significantly reduce the *Candida* spp. colony number in clinical conditions. Given the trend fungal growth in controlled laboratory conditions, their effectiveness in clinical settings may be influenced by short exposure time, interaction with saliva, and OLE formulation type.

As a result of *Candida* spp. colonization of the oral cavity, mucosal changes can be expressed in the form of clinical signs and symptoms or disturbances. Tooyama et al. [34] demonstrated a correlation between the presence of *Candida* spp. in the oral cavity and the appearance of oral signs and symptoms. They reported the development of red oral lesions, dry mouth, and burning tongue related to *Candida* spp. colonization. In this study, we wanted to investigate the impact of OLE in combination with conventional therapy on reducing the incidence of clinical signs and subjective symptoms related to *Candida* spp. colonization.

Numerous studies have proven the antimicrobial and anti-inflammatory effects of OLE [35] primarily in increasing the production of nitric oxide in macrophages, which contributes to the action against microorganisms that cause infections and consequently prevents the development of oral signs and symptoms associated with *Candida* spp. However, in our study, the combination of nystatin or miconazole with OLE did not lead to a significant reduction in clinical signs and symptoms of oral *Candida* infections. Despite OLE’s known antimicrobial and anti-inflammatory properties, its adjunctive use did not enhance the therapeutic effectiveness of conventional antifungal treatments in mucosal symptoms.

Since Interleukin 17 (IL-17A) is one of the key salivary mediators in immune response and protection against *Candida* in the oral cavity [26] previous research by Larussa et al. [36] suggested the effectiveness of oleuropein from OLE in reducing IL-17A expression in patients with ulcerative colitis. Based on these findings, we hypothesized the possible efficacy of OLE on salivary IL-17A. However, the results of our study showed no statistically significant differences in salivary IL-17A levels. The possible effectiveness of OLE on oral cytokine should not be disregarded due to the downward trend of IL-17A in patients who underwent combined therapy compared to conventional therapy.

Although this study provides valuable results on the potential role of OLE as an adjunct to conventional antifungal therapy, the previously mentioned limitations concerning treatment protocol should be acknowledged. All participants received either nystatin or miconazole, with no group receiving only OLE as treatment, which limits our ability to assess OLE independent antifungal effects. A short treatment duration (seven days) may not have been sufficient to observe long-term changes in *Candida* spp. colonization or IL-17A reduction. Additionally, OLE was administered as a mouth rinse, which may have resulted in limited mucosal absorption and reduced efficacy compared to other formulation forms such as gels or systemic use.

Future research should address these limitations by including a study group receiving only OLE, extending the treatment period, and exploring alternative formulations with prolonged mucosal contact. Further studies are needed to clarify OLE’s precise mechanism of action in reducing *Candida* spp. Colonies number, effect on clinical signs and symptoms, and reduction of IL-17A levels.

## 4. Materials and Methods

### 4.1. Participants

The investigation included 59 subjects (n = 59) who attended the Clinic of Dental Medicine, Clinical Hospital Center Rijeka, Croatia. Inclusion criteria required a *Candida* colony greater than 600 CFU/mL determined by the concentrated rinse method and the presence of at least one oral sign or symptom determined through clinical examination. All subjects had their natural teeth restored and/or healthy. Demographic data and medical history were collected at the baseline (zero-day) through anamnesis. Demographic data for all subjects are listed in Table 1. Subjects who had undergone antimicrobial therapy within the previous 30 days, tobacco smokers, patients with systemic diseases, immunologically compromised patients and patients allergic to olive-based products were excluded. Subjects were randomly divided into four groups depending on whether they were treated with OLE + NYS (n = 15), OLE + MIC (n = 15), NYS (n = 14), or MIC (n = 15). The groups were matched for age and gender.

All subjects were informed about the aims and purpose of the investigation and signed the informed consent for participation. This study was approved by both the Ethics Committee of the Clinical Hospital Center Rijeka and the Faculty of Dental Medicine in Rijeka, Croatia.

### 4.2. Clinical Examination

Clinical examination included the assessment of oral mucosal changes—the presence of red lesions on the oral mucosa, red lesions of the tongue, oral dryness, taste disorders, burning sensations in the tongue, and burning sensation of the oral mucosa. The presence of these symptoms was recorded for each subject.

Taste disorders were evaluated by asking the question: “Do you experience any changes in taste?”, with possible answers yes or no. Burning sensations in the tongue and oral mucosa were assessed by asking the questions: “Do you feel a burning sensation on your tongue?” and “Do you feel a burning sensation in your oral mucosa?”, with possible answers yes or no.

The intensity of burning sensations was further evaluated using the Visual Analog Scale (VAS, 0–10), where 0 indicated no burning sensation, and 10 represented the most intense burning sensation.

### 4.3. Sample Collection

The quantity of *Candida* spp. in the oral cavity was determined using the concentrated oral rinse method. For subjects with removable dentures, before sample collection, dentures were removed, disinfected and the oral cavity was rinsed with sterile saline to remove food residue. Subjects were instructed to rinse their mouth with 10 mL of sterile saline, and samples were collected in sterile containers. Each sample was centrifugated at 3500 rpm for 20 min. The supernatant was removed and the resulting deposit was resuspended in 500 µL of sterile saline. A total of 100 µL of the sample was inoculated immediately onto Chromagar *Candida* (CHROMagar, Paris, France) in duplicate. In cases of *Candida* overgrowth, the content was diluted 10 times, and 100 µL of diluted concentrate was immediately inoculated onto Chromagar *Candida* [34]. Plates were incubated at 37 °C for 48 h according to the manufacturer’s guidelines. The agar can differentiate between five species of *Candida* (*C. albicans*, *C. krusei*, *C. glabrata*, *C. tropicalis*, and *Candida* other species). *C. albicans* by growth was identified as light green colored smooth colonies, *C. tropicalis* as blue to metallic blue colored colonies, *C. glabrata* colonies appear as mauve brown smooth colonies, while *C. krusei* appear as pink, fuzzy colonies. Isolates that produced white to mauve colonies were considered for *Candida* other species.

After growth, the colonies were identified and counted, and the number of colonies was expressed as colony-forming units per milliliter (CFU/mL).

Unstimulated saliva samples were collected before treatment (baseline) at the third and seventh day of therapy. Subjects were instructed not to eat or drink for at least 30 min before sample collection. Saliva was collected in the morning between 9 a.m. and 12 p.m. The subjects were instructed to collect the saliva accumulating in their oral cavity using a funnel and a graduated test tube. During saliva collection, subjects were comfortably seated in the dental unit with their heads slightly tilted forward. The saliva collection time was five minutes, and the obtained values were expressed in milliliters per minute. Saliva secretion values of ≥0.36 mL per minute were considered normal salivation, saliva secretion rate in the values of 0.16 to 0.35 mL per minute reduced salivation, and values < 0.15 mL per minute extremely reduced salivation [37].

For cytokine analysis, at least 2 mL of unstimulated saliva was collected before the start of the therapy protocol and on the seventh day after the start of the therapy. Subjects were comfortably accommodated in a dental unit with their head slightly tilted forward. The saliva collection time was five minutes. Saliva was collected in a sterile container and centrifuged at 4000 rpm for 20 min. After centrifugation, the supernatant was stored in a sterile container and frozen at −80 °C. The level of salivary pro-inflammatory cytokine IL-17A was analyzed using the ELISA method (ELISA kit, Diaclone, Besançon, France). IL-17A ELISA was performed according to the manufacturer’s instructions.

### 4.4. Determination of Polyphenols by HPLC

The analysis of olive leaf extract (OLE) polyphenols was performed using high-performance liquid chromatography (HPLC), following the methodology described in our previous study [38]. The dry OLE powder was dissolved in a mixture of methanol and water (8:2), ultrasonicated for 20 min, centrifuged, and filtered through a 0.45 µm cellulose acetate filter. The polyphenolic profile was consistent with previously published results, with oleuropein being the most abundant compound following the latest research on the presence of individual phenolic components in the olive leaf of different varieties [39]. Details on chromatographic conditions and compound identification have been reported in our previous work [38].

### 4.5. Therapy Protocol

Commercial crude OLE (Magdis, Sveta Nedjelja, Croatia) was dissolved in saline at a concentration of 333 mg/mL [35] and stored in bottles for each patient separately. Subjects were instructed to use a graduated pipette to apply 1 mL of the prepared solution, rinse their mouth for 20 s, and spit it out. This process was repeated three times daily for seven days. Ten minutes after OLE application, subjects applied conventional miconazole or nystatin therapy depending on their assigned group. Miconazole (Belupo, Koprivnica, Croatia) therapy was used as an oral gel while Nystatin (Holsten Pharma, Frankfurt, Deutschland) as an oral solution, both according to the manufacturer’s instructions at the same time intervals. They were also instructed not to eat or drink for at least 30 min after the intake therapy.

To evaluate the effect of applied therapy on *Candida* spp., microbiological samples were collected by raising the oral cavity according to the previously described protocol at baseline (before treatment) and third and seventh days. *Candida* colonies were identified, counted, and expressed as CFU/mL. Clinical examination was conducted at the same time intervals to evaluate the effect on oral signs and symptoms and salivation level.

### 4.6. Statistical Analysis

Statistical analysis was performed with MedCalc Statistical Software version 19.1.7 (MedCalc Software Ltd., Ostend, Belgium; https://www.medcalc.org; 2020) and SPSS (IBM Corp. Released 2013. IBM SPSS Statistics for Windows, Version 21.0. Armonk, NY, USA: IBM Corp.). Categorical data were expressed with absolute and relative frequencies. Differences between categorical variables were tested with the χ^2^ test and the Fisher exact test when deemed necessary. Numerical data were expressed as the median and limitations of the interquartile range. Differences in numerical variables between three independent groups were tested with the Kruskal–Wallis test. The level of significance was set to *p* = 0.05.

## 5. Conclusions

This study explored the potential of OLE as an adjunct to standard antifungal therapy in treating oral *Candida* infections. While the addition of OLE did not lead to a significant reduction in *Candida* spp. colony number or salivary IL-17A levels, it showed beneficial effects on oral symptoms. Specifically, OLE combined with nystatin improved salivation rate, while in combination with miconazole alleviated tongue burning. Additionally, a significant reduction in oral mucosa burning was observed in the miconazole group.

The observed downward trend in IL-17A levels in patients receiving OLE suggests a potential immunomodulatory effect that requires further investigation. Our investigation results can help in better understanding the effect of OLE therapy in the treatment of oral *Candida*-related diseases. Considering the complexity of fungal infections and host immune responses, future research should focus on optimizing OLE formulations, exploring its use as a alone therapy, and assessing its long-term impact on oral health.

## Figures and Tables

**Table 1:** Differences in age and gender of patients in relation to groups.

KERRYPNX	Median (Interquartile Range)				*p* *
	MIC	NYS	MIC + OLE	NYS + OLE	
Age(years)	73 (67–82)	71 (63–72)	72 (61–81)	68 (63–80)	0.64
Gender					
F	12	11	10	10	0.85
M	3	3	5	5	

* Kruskal–Wallis test (post hoc Conover).

**Table 2:** Distribution of subjects according to the number of colonies (CFU/100 μL) with regard to the type and period of the applied therapy.

	*p* * Value			
	MIC vs. NYS	MIC vs. MIC + OLE	NYS vs. NYS + OLE	NYS + OLE vs. MIC + OLE
Day 0				
*C. albicans*	0.27	0.68	0.07	0.51
*C. krusei*	0.33	0.80	0.37	0.72
*C. glabrata*	0.44	0.17	0.15	0.32
*C. tropicalis*	>0.99	0.32	0.96	0.32
*C.* other species	>0.99	>0.99	>0.99	>0.99
Day 3				
*C. albicans*	0.97	0.68	0.83	0.70
*C. krusei*	0.74	0.50	0.94	0.89
*C. glabrata*	>0.99	0.32	0.32	>0.99
*C. tropicalis*	>0.99	>0.99	>0.99	>0.99
*C.* other species	>0.99	0.32	>0.99	0.32
Day 7				
*C. albicans*	0.57	0.54	0.28	0.27
*C. krusei*	>0.99	>0.99	0.07	0.07
*C. glabrata*	>0.99	>0.99	>0.99	>0.99
*C. tropicalis*	>0.99	>0.99	>0.99	>0.99
*C.* other species	>0.99	>0.99	>0.99	>0.99

* Mann–Whitney U test.

**Table 3:** Distribution of subjects according to the presence of oral signs and symptoms based on therapy type and treatment duration.

	MIC (n =15)		MIC + OLE (n =15)		p *	NYS (n =14)		NYS + OLE (n =15)		*p* *
	Symptom		Symptom			Symptom		Symptom		
	−	+	−	+		−	+	−	+	
Day 0										
Red lesions of the oral mucosa	4	11	9	6	0.14	7	7	8	7	>0.99
Red lesions of the tongue	14	1	14	1	>0.99	12	2	14	1	0.60
Burning tongue	10	5	8	7	0.71	9	5	11	4	0.70
Dry mouth	3	12	1	14	0.60	2	12	2	13	>0.99
Taste disorder	13	2	15	0	0.48	11	3	15	0	0.09
Burning of the oral mucosa	11	4	11	4	>0.99	13	1	13	2	>0.99
Day 3										
Red lesions of the oral mucosa	5	10	9	6	0.27	7	7	8	7	>0.99
Red lesions of the tongue	14	1	14	1	>0.99	12	2	14	1	0.60
Burning tongue	10	5	9	6	>0.99	9	5	11	4	0.70
Dry mouth	5	10	2	13	0.39	4	10	5	10	>0.99
Taste disorder	13	2	15	0	0.48	11	3	15	0	0.09
Burning of the oral mucosa	11	4	11	4	>0.99	13	1	13	2	>0.99
Day 7										
Red lesions of the oral mucosa	7	8	10	5	0.46	8	6	9	6	>0.99
Red lesions of the tongue	14	1	14	1	>0.99	12	2	14	1	0.60
Burning tongue	12	3	9	6	0.43	9	5	11	4	0.70
Dry mouth	7	8	3	12	0.25	4	10	6	9	0.70
Taste disorder	13	2	15	0	0.48	11	3	15	0	0.09
Burning of the oral mucosa	14	1	14	1	>0.99	13	1	14	1	>0.99

* Fisher exact test.

**Table 4:** Differences in tongue and oral mucosa burning intensity (VAS scale) and salivation rate (mL/min) based on therapy type and duration.

	Median (Interquartile Range)			*p* *
	Day 0	Day 3	Day 7	
Miconazole				
Burning tongue (VAS)	0 (0–3)	0 (0–2)	0 (0–0)	0.007 ^†^
Burning of the oral mucosa (VAS)	0 (0–2)	0 (0–2)	0 (0–0)	0.02 ^†^
Salivation rate	0.5 (0.2–1)	0.6 (0.2–1)	0.6 (0.1–1)	0.09
Nystatin				
Burning tongue (VAS)	0 (0–5)	0 (0–5)	0 (0–4)	0.006 ^‡^
Burning of the oral mucosa (VAS)	0 (0–0)	0 (0–0)	0 (0–0)	0.38
Salivation rate	0.9 (0.4–1)	0.8 (0.3–1)	0.9 (0.3–1)	0.59
Miconazole + OLE				
Burning tongue (VAS)	0 (0–5)	0 (0–5)	0 (0–4)	0.04 ^†^
Burning of the oral mucosa (VAS)	0 (0–8)	0 (0–6)	0 (0–5)	0.09
Salivation rate	0.4 (0.1–0.8)	0.5 (0.1–0.8)	0.5 (0.1–1.0)	0.29
Nystatin + OLE				
Burning tongue (VAS)	0 (0–3)	0 (0–3)	0 (0–3)	0.13
Burning of the oral mucosa (VAS)	0 (0–0)	0 (0–0)	0 (0–0)	0.38
Salivation rate	0.6 (0.1–1.2)	0.8 (0.1–1.1)	0.8 (0.1–1.4)	0.01 ^§^

* Friedman test (Post hoc Conover). ^†^ at the level of *p* < 0.05, a statistically significant reduction in tongue burning and burning of the oral mucosa was observed on day 7 compared to day 0. ^‡^ at the level of *p* < 0.05, a statistically significant reduction in tongue burning was observed on day 7 compared to both day 0 and day 3. ^§^ at the level of *p* < 0.05, a statistically significant increase in salivation was observed on day 7 compared to day 0.

**Table 5:** Differences in IL-17A values (pg/mL) before and after the therapy.

	Median (Interquarile Range)		Difference (95% CI)	*p* *
	Before Therapy	After Therapy		
MIC	15.08 (1.58–19.25)	2.90 (0.83–5.30)	−7.97	0.14
MIC + OLE	5.07 (2.31–9.99)	0.31 (0–6.10)	−3.20 (−15.1–1.76)	0.14
NYS	9.17 (0.91–9.94)	4.09 (1.76–8.39)	−1.66 (−4.7–1.09)	0.93
NYS + OLE	3.38 (0.51–10.52)	2.01 (0.11–13.76)	0.99 (−18.52–6.80)	0.77

* Wilcoxon test.

## Data Availability

The data presented in this study are available on request from the corresponding author. The data are not publicly available due to ethical issues.
